# Clinical aspects of neuroregression: our experience on batten disease

**DOI:** 10.1186/1755-8166-7-S1-I37

**Published:** 2014-01-21

**Authors:** Mahesh Kamate

**Affiliations:** 1 KLES, Belgaum, Karnataka, India

## 

Neuroregression in a child is an important clinical problem faced by a pediatric neurologist. Depending on the initial clinical features they can be broadly divided into grey matter disorders, white matter disorders or combined. The age of onset and the progression of symptoms also help us in further characterization. Involvement of other systems and neuroimaging findings helps us in formulating the differential diagnosis and guides us in the laboratory evaluation and selection of appropriate confirmatory tests. After brief discussion on the clinical approach to neuroregression, here I would like to present our experience with one of the important poliodystrophies in children- Neuronal ceroid lipofuscinosis.

Neuronal ceroid lipofuscinosis is a group of progressive neurodegenerative disorders characterized by accumulation of ceroid lipopigment in lysosomes in neurons and other cell types. Over a period of four years we have diagnosed 20 children with neuronal ceroid lipofucinosis. Of the 20 patients, 5 had infantile type and 15 had late-infantile neuronal ceroid lipofuscinosis. Diagnosis was confirmed by appropriate enzyme assay. Clinical presentation was quite varied. Common presenting features included refractory seizures, developmental delay/regression, and abnormal movements. Visual failure was not common in the present case series, and novel neuroimaging finding in the form of isolated dentate nucleus hyperintensities in PPT related neuronal ceroid lipofuscnioses was noted. During follow-up, all patients had a progressive downhill course and one patient died. Prenatal diagnosis could be offered to one family. Our experience suggests that infantile and late-infantile neuronal ceroid lipofuscinosis is not uncommon in this region of the country and the phenotype is different.

